# Assessing the Risk of QT Prolongation in a Psychiatric Inpatient Cohort: A Retrospective Cross-Sectional Study

**DOI:** 10.3390/ph17101373

**Published:** 2024-10-16

**Authors:** Johan Frederik Mebus Meyer Christensen, Jonathan Hugo Jürgens-Lahnstein, Afrim Iljazi, Stig Ejdrup Andersen, Morten Dahl, Gesche Jürgens

**Affiliations:** 1 Research Unit for Clinical Psychopharmacology, Mental Health Service West, Copenhagen University Hospital—Psychiatry West Region Zealand, 4200 Slagelse, Denmark; gju@regionsjaelland.dk; 2 Department of Clinical Medicine, University of Copenhagen, 2200 Copenhagen, Denmark; afrim.iljazi.04@regionh.dk (A.I.); seja@regionsjaelland.dk (S.E.A.); modah@regionsjaelland.dk (M.D.); 3 Orthopedics Department, Region Central Denmark, Aarhus University Hospital, 8200 Aarhus, Denmark; jonjur@clin.au.dk; 4 Department of Clinical Medicine, Aarhus University, 8200 Aarhus, Denmark; 5 Department of Orthopedic Surgery, Capital Region, University Hospital Copenhagen Rigshospitalet, 2100 Copenhagen, Denmark; 6 Clinical Pharmacology Unit, Zealand University Hospital Roskilde, 4000 Roskilde, Denmark; 7 Department for Clinical Biochemistry, Zealand University Hospital Køge, 4600 Køge, Denmark

**Keywords:** medication review, pharmacotherapy, QT prolongation, adverse-drug reaction, psychotropics, antidepressant agents, antipsychotic agents, drug review

## Abstract

Background: QT prolongation is a potential serious adverse drug reaction, and assessing the risk of QT-prolonging drugs is routinely included in psychotropic medication reviews. However, the actual clinical benefits of such assessments are unknown. We investigate whether QT prolongation (QTc value > 480 ms) manifests in psychiatric inpatients at risk of QT prolongation as identified by assessing drug regimens. Secondly, we test the predictive value of well-known risk factors for QT prolongation. Results: The median patient age was 49 years (IQR 34–64) for patients treated with a median of nine drugs (IQR 6–12) and a median QT-prolonging drug sum of three daily defined dosages (IQR 1.88–4.76). We extracted 290 ECGs for patients where pharmacist-led-medication reviews (PMRs) identified an increased risk of QT prolongation and 190 ECGs for patients with no such risk, identifying 33 cases of verified QT prolongation equally distributed between groups. Unadjusted regression analysis revealed that advanced age (OR 3.27 CI 95% 1.60–6.84) and cardiovascular comorbidity (OR 3.53 CI 95% 1.71–7.29) were associated with manifest QT prolongation, while the QT-prolonging drug load was not. Methods: We reviewed electronic health records (EHRs) of 799 psychiatric inpatients exposed to PMRs made from 1 September 2016 to 31 December 2018 in Region Zealand Denmark. Conclusions: Patients at risk of QT prolongation as identified by drug reviews rarely manifests with actual QT prolongation. Non-pharmacological risk factors seem to be better predictors for identifying patients with QT prolongation.

## 1. Introduction

A pharmacist-led medication review (PMR) is a well-established tool in psychiatry in Denmark to support rational drug prescribing. Not surprisingly, PMRs are often used in geriatric populations, as high proportions of multimorbidity and polypharmacy complicate pharmacological treatment [1]. Studies indicate that PMRs may reduce hospital readmissions and emergency department contacts in elderly inpatients, but whether PMRs also reduce mortality or improve health-related quality of life (QoL) is unknown [2,3]. The benefits of PMRs in psychiatric populations are even less established, despite psychiatric patients sharing many of the pharmacological challenges of geriatric patients such as being exposed to polypharmacy and multimorbidity [4,5]. PMRs may improve psychiatric prescription by enhancing psychotropic deprescribing and medication appropriateness, but the actual clinical benefit, such as reducing the risk of adverse drug-related events, is unknown [1,6]. Furthermore, the degree to which potential pharmacological problems identified by PMRs actually manifest as real problems for the individual patient is unknown.

QT prolongation is a well-described and potential serious adverse reaction to several drug classes, including psychotropics. Generally, the risk increases with increasing dosage and number of QT-prolonging drugs [7]. Thus, it is an important dynamic interaction and routinely included in the revision of drug regimens. Corrected QT (QTc) intervals of >450 ms for men and >470 ms for women are associated with an increased risk of developing serious cardiac arrhythmias such as Torsades de Pointes (TdP) [8,9]. While antiarrhythmics, antipsychotics, antidepressants, and certain antibiotics exert their QT-prolonging effects by directly affecting cardiac repolarization other drugs exert their influence indirectly by affecting circulating levels of electrolytes, thus having a conditional risk of drug-induced QT prolongation [10,11,12,13,14,15]. As the number of drugs associated with QT prolongation is large, an extensive online database listing drugs associated with an increased risk of QT prolongation is available through the CredibleMeds.org © [15]. A repository of drugs with QT-prolonging effects, this database stratifies drugs according to arrhythmic severity, with “known risk of TdP” as the most severe, while also including drugs with a conditional risk of causing QT prolongation [15,16]. However, as non-pharmacological risk factors also contribute to prolonging the QT interval, it is important to identify such factors when assessing the risk of QT prolongation [13]. Well-known risk factors include

- Demographic factors: Age >65 years, female sex, smoking, increased BMI.
- Cardiovascular comorbidity and thyroid disease.
- Electrolytes: hypokalemia, hypomagnesaemia, hypocalcemia and hyponatremia [9,13].
- Substance abuse: cocaine, methadone and a heavy alcohol consumption have been linked to QT prolongation which is particularly relevant for psychiatric patients having a greater proportion of substance abuse compared to the general population [17,18,19,20].
- Chronic inflammation and autoimmune disorders may also increase the risk of QT prolongation [21,22].

The association between non-pharmacological risk factors and QT prolongation is widely reported in several studies. For instance, a large case-report study assessing 61,788 adverse drug reactions from the Swedish national pharmacovigilance database found 88 cases of TdP. Totally, 85% of cases had two or more risk factors associated with QT prolongation with cardiovascular disease, age > 65 years, and female sex as the most common [23]. The primary purpose of this retrospective cross-sectional study was to investigate if QT prolongation manifests in psychiatric inpatients with a potential risk of drug-induced QT prolongation as identified by assessing drug regimens. The second purpose was to test the predictive value of including pharmacological and non-pharmacological risk factors in causing QT prolongation.

## 2. Results

Data originated from psychiatric inpatients who had a PMR made from 1 September 2016 to 31 December 2018. PMRs were conducted on three psychiatric hospitals in Region Zealand Denmark with a total of 405 beds and 8800 annual discharges. Based on the identification of any potential QT-prolonging drugs by PMRs, we divided patients into two groups: the *QT + risk* with an identified risk of drug-induced QT prolongation and *QT ÷ risk* with no identified risk.

Patients in the QT ÷ risk group were older and more often had somatic comorbidities compared to the QT + risk group (Table 1 and Table 2). Generally, psychotropic polypharmacy was common in our cohort, as 35% of patients were prescribed three or more antipsychotics with the QT + risk group in general having a greater degree of psychotropic polypharmacy (Table 3). Evidently, there was no statistical difference in the distribution of patients with verified QT prolongation between groups (QT÷ = 14, QT+ = 19) (Table 4). Patients with verified QT prolongation received a median of 3.89 (IQR 1.63–5.27) cumulative DDDs of QT-prolonging drugs, they were on average 69 years old (IQR 56–79), most were females (61%), and they had an average BMI of 28 cm/kg^2^ (IQR 26–32). Most were non-smokers (55%), and only 9% had a drug or substance abuse. In total, 48% had cardiovascular comorbidity and 55% had an affective disorder. Biochemical test results were normal in this subgroup. At the time of accessing the electronic medical records (fall 2023), 48% of patients were deceased. The unadjusted logistic regression analyses revealed an association between advanced age (OR 3.27 (1.60 to 6.84)) and cardiovascular comorbidity (OR 3.53 (1.71 to 7.29)) and QT prolongation (Table 5). Subsequent sensitivity analysis including excluded dosages of QT-prolonging drugs exceeding five DDDs did not change our results or conclusion (see Supplementary Materials and Section 5).

## 3. Discussion

This study examined how many patients identified as having an increased risk of QT prolongation by reviewing drug regimens manifest with actual QT prolongation by retrospectively reviewing the journals of 799 patients who had a PMR performed as part of a quality development project in Region Zealand in Denmark. When conducting the PRMs, pharmacists used a Danish contemporary guideline incorporating CredibleMeds© (2014), European Medicines Agency (EMA), The US Food and Drug Administration (FDA), the Maudsley prescribing guideline, and Micromedex to identify drugs with QT-prolonging effects [24]. We found no statistical difference in the number of patients with ECG-verified QT prolongation between the group whose prescription pattern predicted an increased risk of QT prolongation and those who did not. Though mean QTc (Bazetts) was 5 ms longer in the QT + group (440 ms CI 95% 437–443) compared to the QT ÷ risk group (435 ms CI 95% 431–438), this was not clinically relevant. Patients with verified QT prolongation were mostly elderly overweight females suffering from cardiovascular comorbidity and an affective disorder. At the time of data extraction (Fall 2023), 48% were deceased, which might be expected considering their advanced age at the time of the PMRs (median age 69 years). As QT prolongation is a rare but potentially serious adverse drug reaction, assessing the risk of potential QT prolongation is routine when reviewing drug regimens. Several studies describe the association between the prolonged QT interval and sudden cardiac death, as well as demonstrate a clear association between an absolute QTc value of >500 ms and an increased risk of arrhythmias [25,26,27,28]. Furthermore, an increase of more than 60 ms after being exposed to any QT-prolonging drug is perceived as dangerous and requires action to reduce the risk of arrhythmias [25]. However, the actual QT-prolonging effect of any single psychotropic drug often ranges between 4 and 20 ms [29,30,31]. Even the highest value is far from the 60 ms, which in absolute terms is considered dangerous. Thus, how are such observations implemented in pharmacological decision-making?

In 2011 and 2012, the FDA reported a dose-dependent association between the selective serotonin reuptake inhibitor (SSRI) citalopram and QT prolongation (up to 18.5 ms) compared to 6–9 ms in sertraline [32]. This led the FDA to decrease the recommended maximum dosage of citalopram from 40 mg to 20 mg in the elderly (>60 years) [33]. This finding has since then been confirmed in several studies. A Danish nationwide case–time–control study estimating the risk of out-of-hospital cardiac arrest in patients treated with specific antidepressants showed an OR of 1.29 (CI 95% 1.02–1.63) in patients treated with citalopram and an OR of 5.14 (CI 95% 2.17–12.2) in patients treated with nortriptyline [34]. Additionally, a Swedish nationwide register-based cohort study found 410 cases of TdP in patients treated with QT-prolonging drugs. Thirty percent were associated with the use of antidepressants (mainly citalopram, mirtazapine, and sertraline) and 17% with antiarrhythmic drugs with an increased risk in the elderly (age > 65 years) [35]. Though citalopram has greater QT-prolonging effects, this may not necessarily be associated with an increased risk of sudden cardiac death nor ventricular arrhythmias [36,37]. Similarly, other psychotropics may only have a limited effect on the QT interval but may be associated with greater mortality. For instance, haloperidol has been reported to have an OR 6.0 (CI 95% 1.50–23.99) unadjusted risk of sudden death but only prolongs the QTc interval by as little as 1.7 ms [30,38]. Thus, it may be non-pharmacological risk factors rather than drugs’ QT-prolonging effects that are indicative of whether drugs have an increased mortality rate. Haloperidol serves as an example, as is it used extensively in somatic hospitals on patients with several concurrent somatic conditions [24].

As the association between non-pharmacological risk factors and QT prolongation is well established, we investigated whether non-pharmacological risk factors were significant or even better predictors for the development of manifest QT prolongation. To quantify the cumulative QT-prolonging drug load, we included all QT-prolonging drugs also found on the CredibleMeds © drug list regardless of arrhythmic severity, as they all contribute to causing QT prolongation [13,16]. Employing unadjusted logistic regression analyses, we found that the presence of cardiovascular comorbidity and age >65 years were stronger predictors of QT prolongation rather than the QT-prolonging drug load. This may be caused by advanced age and cardiovascular comorbidity, leading to myocardial fibrosis, possibly affecting myocardial repolarization and thus the QT interval [39,40]. Furthermore, as the risk of experiencing serotonergic and anticholinergic side effects in response to psychotropic treatment increases in the elderly, it is not surprising to find an increased sensitivity to QT prolongation with advanced age [41].

Contrary to the common belief, we cannot confirm the association between increased QT-prolonging drug load and QT prolongation in this psychiatric cohort. This also means that we cannot confirm any dose-dependent association between QT prolongation and exposure to QT-prolonging drugs. Though our results were surprising, they are supported by an Italian cross-sectional study conducted on psychiatric inpatients, confirming the association between non-pharmacological risk factors and QT prolongation, while psychotropics only played a minor role [42]. Thus, our findings suggest that non-pharmacological risk factors are stronger predictors of QT prolongation than the presence of QT-prolonging drugs in psychiatric inpatients.

Our results have implications for the clinical practice as we urge for a revision of the current way of practicing risk assessments intending to reduce the risk of QT prolongation in psychiatry. Rather than implementing routine drug reviews to reduce the risk of drug-induced QT prolongation, such risk assessment may benefit from focusing on non-modifiable factors (such as advanced age, sex and cardiovascular comorbidity) while addressing modifiable factors (such as correcting hypokalemia, hyponatremia, etc. [43]) when assessing the risk of QT prolongation. By including patients’ medical history, clinical appearance, and laboratory tests in drug reviews, the clinical relevance of such risk assessment may hold greater relevance for prescribing physicians and patients. Additionally, drug reviews may target patients exposed to non-pharmacological risk factors rather than being employed on a routine basis, reducing costs associated with routinely implemented drug reviews. As exposure to QT-prolonging drugs was not indicative of whether patients developed QT prolongation, we imagine that cautious prescribing based on the potential occurrence of rare adverse drug reactions such as QT prolongation can lead clinicians to omit important treatments. Drug reviews routinely implemented to assess the risk of QT prolongation in psychiatric inpatients may in fact harm patients by leading clinicians to refrain from using otherwise effective psychotropic treatments. While certain drugs undoubtedly hold a risk of prolonging the QT interval, clinicians may be advised to focus on the presence on non-pharmacological risk factors rather than the QT-prolonging effects of individual drugs in psychiatric inpatients. Thus, prospective medical decisions cannot rely solely on a list of potential side effects but must rely on a clinical assessment of the individual patient.

## 4. Ethics Statement

This study was registered on the record of scientific research projects in Region Zealand (registration number 084-2022, final approval date 23 November 2023). Permission to access the electronic health records (EHRs) was obtained from the Team for Patient Record Data and the Regional Council (EMN-2023-00985, final approval date 16 November 2023). 

## 5. Methods

PMRs were implemented to improve drug prescribing in psychiatric inpatients as part of a quality development project. Patients qualified for a PMR if exposed to

- ≥5 fixed scheduled drugs;
- ≥2 fixed scheduled antipsychotics
  and/or;
- Fixed scheduled antidepressants;
- Fixed scheduled treatment with an antipsychotic and a benzodiazepine without an end-date;
- ≥1 drug with known high risk of arrhythmias (tricyclic antidepressants, lithium, valproate, and clozapine).

PMRs were conducted to support a physician-led medication review and were entirely based on patients’ EHRs (Supplementary material). In total, 905 PMRs had been conducted, 106 of which were repeated instances of already included patients and were excluded, leaving 799 PMRs for the analyses. For patients with multiple PMRs, only the most recent was included, since biochemical test results were only accessible for the patient ID matching the last PMR. Complementary clinical data were extracted from the EHRs but were not available in their entirety for all included patients. The PMRs’ summaries included information on name, patient ID, drug regimen, and exposure to any QT-prolonging drugs. QT-prolonging drugs were coded according to a contemporary existing Danish guideline also incorporating a contemporary version of the CredibleMeds.org © list of QT-prolonging drugs [24].

Clinical data were recorded using RedCap (version 13.7.14). A custom Python script (version 3.8) extracted Anatomical Therapeutical Chemical codes (ATC) from a Danish online repository on drugs marketed in Denmark [44] (January 2024). Based on the ATC codes, the Python script extracted the defined daily dose (DDD) with units from the Norwegian Institute of Public Healths ATC/DDD Index [45] (January 2024) for all drugs in the drug regimens. Using the dose and number of daily administrations, the total daily dosages (TDDs) were calculated. For drugs administered as needed, the TDDs were calculated based on the maximum number of allowed daily administrations. For as-needed prescriptions with missing information on the maximum, the maximum number of daily administrations was equal to one. For depot injections and long-acting agents, the average daily dosage was calculated by dividing the dosage by the dosage intervals (e.g., 400 mg every 21 days: 400/21 = 19 mg as daily dosage). For each patient, we calculated a composite cumulative daily DDD dosage of all QT-prolonging drugs found on CredibleMeds© (2024). Individual QT-prolonging drugs with a DDD > 5 were excluded as outliers.

We extracted height, weight, body mass index (BMI), age at time of PMR, sex (as stated in the EHRs), information on any potential drug or substance abuse, smoking status, whether patients were alive when extracting data (Fall 2023), comorbidities according to the International Classification of Diseases (ICD-10), blood pressure, and pulse measurements from the EHRs. We were unable to characterize patients’ substance abuse in detail, making us unable to include these in our logistic regression analyses. ECGs were extracted from 480 patients collected 1 month prior to and 1 month after the date of the PMR. We included the autogenerated heart frequency and QTc intervals using Fridericias and Bazetts formula. We defined QT prolongation as a QTc interval >480 ms regardless of sex or mode of calculation [25]. We extracted biochemical test results collected from 1 September 2016 to 31 January 2019 for 494 patients. We used only the ECG recording and biochemical test results closest to the date of the PMR and in instances with more than one ECG recording or biochemical test result available at the specific date a mean of the values were estimated. We were able to include the following routinely collected biochemical analyses: potassium, sodium, total-calcium, alkaline phosphatase, cholesterol, thyroid stimulating hormone (TSH), Alanine Aminotransferase (ALAT), C-reactive protein (CRP), creatinine and magnesium. However, we were only able to extract sufficient data for potassium, sodium, total calcium, and alkaline phosphatases to be included in the logistic regression analyses.

## 6. Statistical Analyses

We compared the average QTc values and the distribution of patients with verified QT prolongation between groups using the Welch Two-Sample *t*-test for parametric testing and the Pearson’s Chi-squared test for categorical variables. We employed a Wilcoxon rank sum test for nonparametric testing. Results were reported with 95 confidence intervals (95% CI) and medians with interquartile range (IQR). A two-tailed *p* < 0.05 was considered statistically significant. R for statistical computing (version 4.3.3) was used for the analyses [46]. To test the predictive value of including both pharmacological and non-pharmacological risk factors when assessing the risk of QT prolongation, we employed unadjusted and adjusted logistic regression analysis using mainly dichotomized outcome variables. We included the following well-established risk factors in our analysis [9,13]:

- Age >65 years;
- Being female;
- Smoking;
- Increased BMI (>25 cm/kg^2^);
- Having a cardiovascular comorbidity;
- Hypokalemia (<3.5 mmol/L);
- Hypocalcemia (<2.20 mmol/L);
- Hyponatremia (<135 mmol/L);
- High alkaline phosphatases (>1.75 µkat/L);
- Hypertension (systolic blood pressure >135 mmHg);
- The use of QT-prolonging drugs according to CredibleMeds© (not dichotomized).

## 7. Limitations

We extracted ECGs for 480 patients with some clinical data missing, making us unable to make definitive conclusions based on our findings. Having insufficient data on inflammatory biomarkers meant we were unable to adjust for inflammation in analyses. Furthermore, as we were unable to characterize patients’ substance abuse (such as the type of agent being abused), while also missing information on 220 of 799 patients, we were also unable to adjust for substance abuse in our analyses. Additionally, the QT + risk group may only be administered QT-prolonging drugs as they lack non-pharmacological risk factors otherwise associated with QT prolongation. Since patients in the QT ÷ risk group were exposed to several non-pharmacological risk factors, physicians may already have optimized pharmacological treatment prior to the PMRs, reducing the risk of drug-induced QT prolongation affecting the results of our study. As we lack longitudinal data, the potential preventive effect of PMRs in reducing the risk of future instances of QT prolongation is not included in our study. Furthermore, as the results from the unadjusted logistic regression model cannot be replicated in our adjusted logistic regression our results were prone to uncertainty. Lastly, as this study only includes psychiatric inpatients from Region Zealand Denmark, the generalizability of our results may not be applicable to other patient cohorts.

## 8. Conclusions

We found no association between patients identified as having a potential increased risk of QT prolongation based on their current drug regimens and patients with actual QT prolongation in a psychiatric inpatient cohort. Patients with actual QT prolongation were mainly elderly, overweight females with an affective disorder and, in 48% of cases, cardiovascular comorbidity. In an unadjusted logistic regression analysis, we confirmed the association between advanced age and cardiovascular comorbidity and QT prolongation. Surprisingly, we could not confirm the association between increased QT-prolonging drug load and QT prolongation. Thus, a potential risk of QT prolongation as identified by assessing drug regimens rarely manifests as actual QT prolongation in psychiatric inpatients. As non-pharmacological risk factors are stronger predictors of QT prolongation rather than exposure to QT-prolonging drugs, we encourage greater focus on non-pharmacological risk factors when assessing the risk of QT prolongation in psychiatric inpatients.

## Figures and Tables

**Table 1:** Demographics.

Characteristic	N ^1^	All Patients	QT ÷ Risk (N = 316)	QT + Risk (N = 483)	*p*-Value ^2^
Age, Median (IQR)	774	49 (34–64)	55 (40–72)	45 (32–58)	<0.001
Unknown		25	8	17	
Gender, n (%)	686				0.34
Female		343 (43)	136 (43)	207 (43)	
Male		343 (43)	142 (45)	201 (42)	
Unknown		113 (14)	38 (12)	75 (16)	
Height (centimeters), Median (IQR)	564	171 (165–180)	171 (164–180)	172 (165–180)	0.20
Unknown		235	95	140	
Weight (kilograms), Median (IQR)	382	80 (66–94)	78 (62–92)	80 (66–96)	0.13
Unknown		417	157	260	
BMI (cm/kg^2^), Median (IQR)	382	27 (23–31)	26 (23–30)	27 (23–32)	0.27
Unknown		417	157	260	
Smoker, n (%)	574				0.087
No		212 (27)	92 (29)	120 (25)	
Yes		362 (45)	128 (41)	234 (48)	
Unknown		225 (28)	96 (30)	129 (27)	
Drug or substance abuse, n (%)	579				0.62
No		406 (51)	163 (52)	243 (50)	
Yes		173 (22)	63 (20)	110 (23)	
Unknown		220 (28)	90 (28)	130 (27)	
Alive at present day, n (%)	689				<0.001
No		143 (21)	77 (28)	66 (16)	
Yes		546 (79)	202 (72)	344 (84)	
Unknown		110	37	73	
Diastolic blood pressure (mmHg), Median (IQR)	348	84 (76–93)	85 (76–94)	83 (75–91)	0.12
Unknown		451	172	279	
Systolic blood pressure (mmHg), Median (IQR)	348	136 (122–148)	139 (125–154)	133 (121–145)	0.009
Unknown		451	172	279	

^1^ Number of available observations. ^2^ Wilcoxon rank sum test; Pearson’s Chi-squared test.

**Table 2:** The distribution of comorbidities in the study cohort according to ICD-10.

Characteristic	All Patients, N = 799	QT ÷ Risk (N = 316)	QT + Risk (N = 483)	*p*-Value ^1^
Affective disorder, n (%)	180 (23)	85 (27)	95 (20)	0.017
Cancer, n (%)	17 (2.1)	13 (4.1)	4 (0.8)	0.002
Cardiovascular comorbidity, n (%)	125 (16)	63 (20)	62 (13)	0.007
Diabetes, n (%)	48 (6.0)	26 (8.2)	22 (4.6)	0.033
Nephrological disease, n (%)	9 (1.1)	5 (1.6)	4 (0.8)	0.33
Pacemaker, n (%)	10 (1.3)	6 (1.9)	4 (0.8)	0.21
Thyroid disease, n (%)	29 (3.6)	15 (4.7)	14 (2.9)	0.17
Psychotic disorder, n (%)	188 (24)	53 (17)	135 (28)	<0.001
^2^ ADHD, n (%)	29 (3.6)	10 (3.2)	19 (3.9)	0.57
Anxiety, n (%)	56 (7.0)	35 (11)	21 (4.3)	<0.001
Total number of diseases, Median (IQR)	2 (1–3)	2 (1–4)	2 (2–3)	0.43
Unknown	320	121	199	

^1^ Pearson’s Chi-squared test; Fisher’s exact test; Wilcoxon rank sum test. ^2^ Attention-Deficit/Hyperactivity Disorder.

**Table 3:** Drug use according to daily defined dosages (DDD) and ATC codes.

Characteristic	N	All Patients, N = 799	QT ÷ Risk, N = 316	QT + Risk, N = 483
Number of prescription drugs, Median (IQR)	799	9 (6–12)	8 (5–11)	9 (6–12)
Cummulative DDDs of QT-prolonging drugs according to CredibleMeds© (2024), Median (IQR)	739	3.00 (1.88–4.76)	2.19 (1.25–3.68)	3.75 (2.28–5.33)
Patients with no data		60	26	34
Number of psychotropic drugs associated with QT prolongation according to CredibleMeds© (2024), n (%)	765			
1		137 (18)	112 (39)	25 (5.2)
2		210 (27)	100 (35)	110 (23)
3		211 (28)	51 (18)	160 (33)
4		122 (16)	14 (4.9)	108 (23)
5		54 (7.1)	5 (1.8)	49 (10)
6		16 (2.1)	0 (0)	16 (3.3)
7		11 (1.4)	2 (0.7)	9 (1.9)
8		3 (0.4)	1 (0.4)	2 (0.4)
11		1 (0.1)	0 (0)	1 (0.2)
Patients with no data		34	32	2
Number of somatic drugs associated with QT prolongation according to CredibleMeds© (2024), n (%)	386			
1		229 (59)	99 (61)	130 (58)
2		98 (25)	37 (23)	61 (27)
3		44 (11)	21 (13)	23 (10)
4		10 (2.6)	5 (3.1)	5 (2.2)
5		4 (1.0)	0 (0)	4 (1.8)
6		1 (0.3)	0 (0)	1 (0.4)
Patients with no data		413	155	258
Cummulative DDDs of antidepressants (N06A), Median (IQR)	369	1.50 (1.00–2.25)	1.50 (1.00–2.38)	1.50 (1.00–2.25)
Patients with no data		430	181	249
Cummulative DDDs of antiepileptics (N03A), Median (IQR)	258	0.67 (0.38–1.00)	0.67 (0.40–1.00)	0.67 (0.33–1.00)
Patients with no data		541	230	311
Cummulative DDDs of antipsychotics (N05A, including lithium), Median (IQR)	665	1.78 (0.75–3.00)	1.17 (0.50–2.25)	2.02 (1.00–3.38)
Patients with no data		134	98	36
Cummulative DDDs of anxiolytics incl. benzodiazepines (N05B), Median (IQR)	139	1.5 (0.9–3.5)	1.5 (0.9–5.5)	1.3 (0.7–2.7)
Patients with no data		660	269	391
Cummulative DDDs of hypnotics excl. benzodiazepines (N05C), Median (IQR)	195	1.00 (1.00–2.00)	1.00 (1.00–1.50)	1.00 (1.00–2.00)
Patients with no data		604	229	375
Number of Antidepressants (N06A), n (%) patients are exposed to	369			
0		430 (54)	181 (57)	249 (52)
1		256 (32)	94 (30)	162 (34)
2		97 (12)	39 (12)	58 (12)
3		14 (1.8)	2 (0.6)	12 (2.5)
4		2 (0.3)	1 (0.3)	1 (0.2)
Number of Antiepileptics (N03A), n (%), n (%) patients are exposed to	258			
0		540 (68)	230 (73)	310 (64)
1		184 (23)	56 (18)	128 (27)
2		55 (6.9)	25 (7.9)	30 (6.2)
3		19 (2.4)	6 (1.9)	13 (2.7)
4		1 (0.1)	0 (0)	1 (0.2)
Number of Antipsychotics (N05A, including lithium), n (%) patients are exposed to	690			
0		109 (14)	92 (29)	17 (3.5)
1		169 (21)	106 (33)	63 (13)
2		244 (31)	78 (25)	166 (34)
3		161 (20)	32 (10)	129 (27)
4		74 (9.3)	4 (1.3)	70 (15)
5		29 (3.6)	2 (0.6)	27 (5.6)
6		5 (0.6)	0 (0)	5 (1.0)
7		7 (0.9)	3 (0.9)	4 (0.8)
11		1 (0.1)	0 (0)	1 (0.2)
Number of Hypnotics excl. benzodiazepines (N05C), n (%) patients are exposed to	196			
0		604 (76)	229 (72)	375 (78)
1		173 (22)	77 (24)	96 (20)
2		19 (2.4)	9 (2.8)	10 (2.1)
3		2 (0.3)	1 (0.3)	1 (0.2)
4		1 (0.1)	1 (0.3)	0 (0)
Number of Anxiolytics incl. benzodiazepines (N05B), n (%) patients are exposed to	144			
0		655 (82)	268 (85)	387 (80)
1		121 (15)	37 (12)	84 (17)
2		17 (2.1)	8 (2.5)	9 (1.9)
3		6 (0.8)	4 (1.3)	2 (0.4)

**Table 4:** QTc intervals and manifest QT prolongation between groups.

Characteristic	Patients Included:	All Patients (N = 799)	95% CI ^1^	QT ÷ Risk (N = 316)	95% CI ^1^	QT + Risk (N = 483)	95% CI ^1^	*p*-Value ^2^
Mean Pulse (beats per minute) (SD ^3^, Minimum, Maximum)	480	80 (15, 43, 127)	79, 82	78 (14, 43, 127)	76, 80	82 (15, 49, 126)	80, 84	0.008
Unknown Mean Fridericia (milliseconds) (SD, Minimum, Maximum)	480	319 419 (24, 363, 506)	417, 421	127 418 (26, 363, 506)	414, 421	192 419 (22, 366, 486)	417, 422	0.4
Unknown Mean Bazetts (ms) (SD, Minimum, Maximum)	480	319 438 (25, 354, 520)	436, 440	127 435 (27, 354, 520)	431, 438	192 440 (24, 382, 508)	437, 443	0.031
Unknown Number of patients with QTc > 480 ms n/N(%) Unknown	480	319 33/480 (6.9%) 319	4.8%, 9.6%	127 14/190 (7.4%) 127	4.2%, 12%	192 19/290 (6.6%) 192	4.1%, 10%	0.7

^1^ CI = 95% Confidence Interval. ^2^ Welch Two Sample *t*-test; Pearson’s Chi-squared test. ^3^ Standard deviation.

**Table 5:** Risk factors for QT prolongation.

	Unadjusted			Adjusted	
Characteristic	All Patients = 480 ^1^	OR (95% CI) ^2^	*p*-Value	OR (95% CI) ^2^	*p*-Value
Female	480	1.43 (0.70 to 3.00)	0.34	1.86 (0.38 to 11.3)	0.46
Elderly (>65 years)	480	3.27 (1.60 to 6.84)	0.001	1.84 (0.33 to 13.0)	0.50
Cummulative DDDs of QT prolonging agents according to CredibleMeds© 2024	437	1.06 (0.90 to 1.24)	0.45	1.22 (0.83 to 1.79)	0.30
Hyponatremia (<135 mmol/L)	422	2.39 (0.77 to 6.25)	0.10	4.36 (0.51 to 29.4)	0.14
Hypocalcemia (<2.20 mmol/L)	375	1.46 (0.41 to 4.06)	0.50	1.92 (0.09 to 16.2)	0.59
High alkaline phosphatase (>1.75 µkat/L)	409	1.48 (0.53 to 3.54)	0.42	1.90 (0.23 to 11.5)	0.50
Hypertension (Systolic BP >135 mmHg)	310	0.56 (0.22 to 1.34)	0.20	0.68 (0.12 to 3.24)	0.64
Smoker	452				
No		Ref		Ref	
Yes		0.50 (0.24 to 1.04)	0.064	0.36 (0.04 to 2.55)	0.32
Cardiovascular comorbidity	480	3.53 (1.71 to 7.29)	<0.001	1.83 (0.38 to 8.95)	0.44
Hypokalemia (<3.5 mmol/L)	419	0.48 (0.08 to 1.65)	0.32	0.29 (0.01 to 2.29)	0.32
Overweight (BMI > 25 cm/kg^2^)	308	2.62 (0.93 to 9.32)	0.092	6.73 (1.03 to 138)	0.094

^1^ Number of observations in each variable. ^2^ OR = Odds Ratio, CI = 95% Confidence Interval, AIC = 78.

## Data Availability

The datasets presented in this article are not readily available because of ethical concerns. Parts of the data (e.g., code) may be available. Requests should be directed to J.C.

## Supplementary Materials

The following supporting information can be downloaded at: https://www.mdpi.com/article/10.3390/ph17101373/s1.
