# Supplementary material for: Assessing the Risk of QT Prolongation in a Psychiatric Inpatient Cohort: A Retrospective Cross-Sectional Study
Source: Pharmaceuticals (Basel). 2024 Oct 16;17(10):1373. doi: 10.3390/ph17101373 (PMC11510743; doi:10.3390/ph17101373)
Supplement: Supplementary file 1 [file pharmaceuticals-17-01373-s001.zip › pharmaceuticals-3233045-supplementary.pdf]

Supplementary materials

Supplemental results (sensitivity analysis) including excluded QT prolonging drugs with a DDD > 5:

Regarding table 3:

Median cumulative DDDs of QT prolonging drugs according to CredibleMeds© (2024) (IQR):

- **QT ÷ risk:** 2.34 (1.33, 3.68)
- **QT + risk:** 4.00 (2.50, 5.88)

Regarding table 5:

- Female: Unadjusted OR 1.43 (95 % CI 0.70 to 3.00) p value 0.34. Adjusted OR 1.87 (95 % CI 0.37 to 11.3) p value 0.46.
- Elderly: Unadjusted OR 3.27 (95 % CI 1.60 to 6.84) p value <**0.001**. Adjusted OR 1.65 (95 % 0.28 to 12.1) p value 0.46.
- Cummulative DDDs of QT prolonging agents according to CredibleMeds © 2024: Unadjusted OR 1.02 (95 % CI 0.88 to 1.16) p value 0.79. Adjusted OR 1.22 (95 % CI 0.92 to 1.64) p value 0.16.
- Hyponatremia: Unadjusted OR 2.39 (95 % CI 0.77 to 6.25) p value 0.10. Adjusted OR 4.75 (95 % CI 0.55 to 32.3) p value 0.12.
- Hypocalcemia: Unadjusted OR 1.46 (95 % CI 0.41 to 4.06) p value 0.50. Unadjusted OR 2.22 (95 % CI 0.10 to 20.0) p value 0.52.
- High alkaline phosphatasis: Unadjusted OR 1.48 (95 % CI 0.53 to 3.54) p value 0.42. Adjusted OR 1.71 (95 % CI 0.19 to 10.7) p value 0.58.
- Hypertension: Unadjusted OR 0.56 (95 % CI 0.22 to 1.34) p value 0.20. Adjusted OR 0.70 (0.12 to 3.40) p value 0.66.

- Smoker (yes): Unadjusted OR 0.50 (95 % CI 0.24 to 1.04) p value 0.064. Adjusted OR 2.03 (95 5 CI 0.41 to 10.6) p value 0.29
- Cardiovascular comorbidity: Unadjusted OR 3.53 (95 % CI 1.71 to 7.29) p value **<0.001**. Adjusted OR 2.03 (95 % CI 0.41 to 10.6) p value 0.38.
- Hypokalemia: Unadjusted OR 0.48 (95 % CI 0.08 to 1.65) p value 0.32. Adjusted OR 0.31 (95 % CI 0.01 to 2.46) p value 0.34.
- Obesity: Unadjusted OR 2.62 (95 % CI 0.93 to 9.32) p value 0.092. Adjusted OR 6.12 (95 % CI 0.90 to 127) p value 0.12.

Supplemental references:

The Danish Health Authorities (2019): https://www.sst.dk/-/media/udgivelser/2020/evaluering-af-tvaerfaglige-medicinteams-i-psykiatrien Accessed May 2024 (Danish)

Analyses were conducted using the R Statistical language (version 4.3.3; R Core Team, 2024) on Windows 10 x64 (build 19045), using the packages flextable (version 0.9.5; Gohel D, Skintzos P, 2024), lubridate (version 1.9.3; Grolemund G, Wickham H, 2011), report (version 0.5.8; Makowski D
et al., 2023), tibble (version 3.2.1; Müller K, Wickham H, 2023), gtsummary (version 1.7.2; Sjoberg D et al., 2021), ggplot2 (version 3.5.0; Wickham H, 2016), forcats (version 1.0.0; Wickham H, 2023), stringr (version 1.5.1; Wickham H, 2023), tidyverse (version 2.0.0; Wickham H et al., 2019), readxl (version 1.4.3; Wickham H, Bryan J, 2023), dplyr (version 1.1.4; Wickham H et al., 2023), purrr (version 1.0.2; Wickham H, Henry L, 2023), readr (version 2.1.5; Wickham H et al., 2024) and tidyr (version 1.3.1; Wickham H et al., 2024).

References

- Gohel D, Skintzos P (2024). _flextable: Functions for Tabular Reporting_. R package version 0.9.5, <https://CRAN.R-project.org/package=flextable>.
- Grolemund G, Wickham H (2011). “Dates and Times Made Easy with lubridate.” _Journal of Statistical Software_, *40*(3), 1-25. <https://www.jstatsoft.org/v40/i03/>.
- Makowski D, Lüdecke D, Patil I, Thériault R, Ben-Shachar M, Wiernik B (2023). “Automated Results Reporting as a Practical Tool to Improve Reproducibility and Methodological Best Practices Adoption.” _CRAN_. <https://easystats.github.io/report/>.
- Müller K, Wickham H (2023). _tibble: Simple Data Frames_. R package version 3.2.1, <https://CRAN.R-project.org/package=tibble>.
- R Core Team (2024). _R: A Language and Environment for Statistical Computing_. R Foundatio n for
Statistical Computing, Vienna, Austria. <https://www.R-project.org/>.
- Sjoberg D, Whiting K, Curry M, Lavery J, Larmarange J (2021). “Reproducible Summary Tables with
the gtsummary Package.” _The R Journal_, *13*, 570-580. doi:10.32614/RJ-2021-053 <https://doi.org/10.32614/RJ-2021-053>, <https://doi.org/10.32614/RJ-2021-053>.

- Wickham H (2016). _ggplot2: Elegant Graphics for Data Analysis_. Springer-Verlag New York. I
SBN
978-3-319-24277-4, <https://ggplot2.tidyverse.org>.
- Wickham H (2023). _forcats: Tools for Working with Categorical Variables (Factors)_. R packag
e
version 1.0.0, <https://CRAN.R-project.org/package=forcats>.
- Wickham H (2023). _stringr: Simple, Consistent Wrappers for Common String Operations_. R
package version 1.5.1, <https://CRAN.R-project.org/package=stringr>.
- Wickham H, Averick M, Bryan J, Chang W, McGowan LD, François R, Grolemund G, Hayes A, H
enry L,
Hester J, Kuhn M, Pedersen TL, Miller E, Bache SM, Müller K, Ooms J, Robinson D, Seidel DP, Spi
nu
V, Takahashi K, Vaughan D, Wilke C, Woo K, Yutani H (2019). “Welcome to the tidyverse.” _Journ
al
of Open Source Software_, *4*(43), 1686. doi:10.21105/joss.01686
<https://doi.org/10.21105/joss.01686>.
- Wickham H, Bryan J (2023). _readxl: Read Excel Files_. R package version 1.4.3,
<https://CRAN.R-project.org/package=readxl>.
- Wickham H, François R, Henry L, Müller K, Vaughan D (2023). _dplyr: A Grammar of Data
Manipulation_. R package version 1.1.4, <https://CRAN.R-project.org/package=dplyr>.
- Wickham H, Henry L (2023). _purrr: Functional Programming Tools_. R package version 1.0.2,
<https://CRAN.R-project.org/package=purrr>.
- Wickham H, Hester J, Bryan J (2024). _readr: Read Rectangular Text Data_. R package version
2.1.5, <https://CRAN.R-project.org/package=readr>.
- Wickham H, Vaughan D, Girlich M (2024). _tidyr: Tidy Messy Data_. R package version 1.3.1,
<https://CRAN.R-project.org/package=tidyr>.

Study data were collected and managed using REDCap electronic data capture tools hosted at Region Zealand, Denmark[1,2] REDCap (Research Electronic Data Capture) is a secure, web-based software platform designed to support data capture for research studies, providing 1) an intuitive interface for validated data capture; 2) audit trails for tracking data manipulation and export procedures; 3) automated export procedures for seamless data downloads to common statistical packages; and 4) procedures for data integration and interoperability with external sources.

References (RedCap)

[1]PA Harris, R Taylor, R Thielke, J Payne, N Gonzalez, JG. Conde, Research electronic data capture (REDCap) – **A metadata-driven methodology and workflow process for providing translational research informatics support**, *J Biomed Inform. 2009 Apr;42(2):377-81.*

[2]PA Harris, R Taylor, BL Minor, V Elliott, M Fernandez, L O’Neal, L McLeod, G Delacqua, F Delacqua, J Kirby, SN Duda, REDCap Consortium, **The REDCap consortium: Building an international community of software partners**, *J Biomed Inform. 2019 May 9 [doi: 10.1016/j.jbi.2019.103208]*

Python packages employed:

Pandas (version 2.2.1): Data structures for statistical computing in python, McKinney, Proceedings of the 9th Python in Science Conference, Volume 445, 2010.

BeautifulSoup (version 4.12.3).

Numpy (version 1.26.0): Harris, C.R., Millman, K.J., van der Walt, S.J. et al. *Array programming with NumPy*. Nature 585, 357–362 (2020). DOI: 10.1038/s41586-020-2649-2
